# How Gardening in Detroit Influences Physical and Mental Health

**DOI:** 10.3390/ijerph19137899

**Published:** 2022-06-28

**Authors:** Alyssa W. Beavers, Ashley Atkinson, Lauren M. Varvatos, Mary Connolly, Katherine Alaimo

**Affiliations:** 1 Department of Food Science and Human Nutrition, Michigan State University, G.M. Trout FSHN Building, 469 Wilson Rd #204, East Lansing, MI 48824, USA; beavers.alyssa@wayne.edu (A.W.B.); lauren.varvatos@med.wayne.edu (L.M.V.); connmary@med.umich.edu (M.C.); 2 Department of Nutrition and Food Science, Wayne State University, Science Hall, 5045 Cass Ave #3225, Detroit, MI 48201, USA; 3 Keep Growing Detroit, Detroit, 1445 Adelaide St, Detroit, MI 48207, USA; ashley@keepgrowingdetroit.org; 4 School of Medicine, Wayne State University, 540 E Canfield St, Detroit, MI 48201, USA; 5 Pediatric Diabetes, University of Michigan C.S. Mott Children’s Hospital, 1540 E Hospital Dr, Ann Arbor, MI 48109, USA

**Keywords:** mental health, gardening, physical health, urban gardening, urban health

## Abstract

Gardening has the potential to improve health by providing access to nature, vegetables, and physical activity. However, scarce research exists on the health impacts of gardening on racial and ethnic minority urban populations in the United States. This study used qualitative interviews to examine the perceived physical and mental health effects of gardening in a primarily African American sample of 28 gardeners. Prominent physical health impacts attributed to gardening included providing an enjoyable source of activity, management of chronic diseases, and improved physical functioning. Participants also reported that gardening improved their mood, relieved stress, was an important part of their spirituality, contributed to their personal growth, and provided an opportunity for helping others. These findings suggest that gardening may improve physical and mental health among diverse groups.

## 1. Introduction

The vast majority of American adults do not meet physical activity or diet recommendations [1,2], which increases their risk of chronic diseases, such as heart disease and diabetes [3,4,5,6]. Additionally, mental disorders, such as anxiety disorders, depression, and others, affect around one in five American adults annually [7]. Urban gardening offers a holistic, place-based strategy to reduce the risk of chronic diseases and improve mental wellbeing by increasing access to fresh produce and providing a nature-based place to be physically active [8]. Observational studies have found that gardeners consume fruits and vegetables more often than non-gardeners [9,10,11], and small or pilot gardening intervention studies have demonstrated increased intake of fruits and vegetables after gardening [12,13,14]. Gardening is a source of moderate to vigorous physical activity for younger adults, and low to moderate physical activity for older adults [15,16]; thus, gardening can be used to help meet the recommendation of at least 150 min of moderate to vigorous physical activity weekly [17]. However, evidence for gardening as an intervention to increase physical activity levels is lacking. For example, two studies have found that gardeners and non-gardeners have similar levels of activity [18,19]. Another study found that gardeners aged 62 and older were more active in the summer than non-gardeners of the same age, but there was no difference in activity level between gardeners and non-gardeners under the age of 62 [20].

The health benefits of gardening extend beyond physical health. Several studies have demonstrated that gardening is associated with reductions in stress and other elements of mental wellbeing [19,20,21]. One potential mechanism for this relationship is through exposure to nature offered by gardening. Exposure to nature, such as parks, forests, or more green space near the home, is associated with a variety of mental wellbeing outcomes, including reductions in stress and depression [22,23]. Gardeners are exposed to nature through tending to their garden, and by passively experiencing the sights and sounds of living things (i.e., birds, insects, and plants) while in the garden. There is mounting evidence linking nature exposure to other health outcomes as well, such as increased physical activity, lower blood pressure, and reduced mortality [23,24]. Nature exposure can be infrequent in urban areas, which is attracting growing concern as urbanization increases, and gardening provides an easily accessible form of urban nature.

In addition to the previously described quantitative studies, qualitative studies on gardening and health allow insights into the lived experiences of gardeners, and can unearth the perceived mental and physical health effects of urban gardening. These studies have found that gardeners commonly perceive that physical activity, diet, and mental wellbeing are promoted by gardening [25,26,27,28,29,30]. While this research has been conducted in several Western countries (the U.K. [27], Canada [28,29], Australia [26], and the US), only a few of these studies have been conducted in the US [25,30]. Additionally, most of the previous qualitative research that examined the relationship between gardening and health was conducted with all white [26,27] or mostly white [25] participants, or did not provide demographic information about the participants [28,29]. To elucidate the potential role of gardening in improving health in diverse populations, there is a need to include racially and ethnically diverse samples in this line of research. The aim of this study was to qualitatively explore the perceived impacts of gardening on physical activity, physical health, and mental wellbeing in a primarily African American sample of urban gardeners in Detroit, Michigan.

## 2. Materials and Methods

### 2.1. Community Partner Description

**Figure 1:**
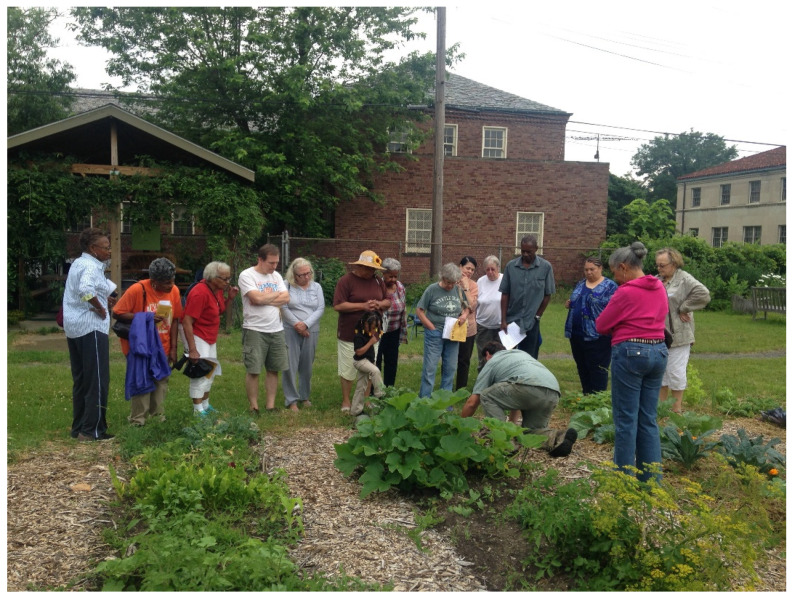
Garden Resource Program members attending a gardening class.

This research was conducted in collaboration with Keep Growing Detroit (KGD), a non-profit organization serving gardeners in the cities of Detroit, Hamtramck, and Highland Park, Michigan. In 2021, Keep Growing Detroit supported approximately 24,088 Detroiters in 2020 gardens with their Garden Resource Program. In contrast to many other gardening organizations, Keep Growing Detroit does not manage or oversee their member’s gardens. Rather, they support gardens initiated by individuals or community organizations by providing material resources (i.e., seeds, plants, and compost), technical support, and gardening education (Figure 1). Keep Growing Detroit also offers a community garden leadership course, a market garden incubation program, and hosts city-wide events for their gardeners.

**Figure 2:**
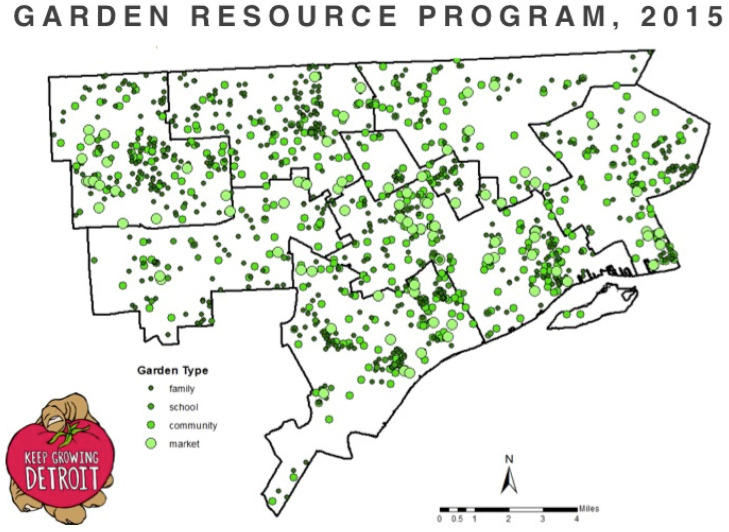
Location and type of Garden Resource Program member gardens.

While many gardening organizations serve either home gardeners or community gardeners, Keep Growing Detroit serves any type of gardener located in their service area. Their member gardens are located throughout the city of Detroit (Figure 2), and include community gardens, which they define as gardens cultivated by gardeners from more than one family; family gardens, which they define as gardens where one or more members within a single family garden; school gardens located at schools or early child-education centers; and market gardens, where produce is grown primarily for sale. Most community gardens in Detroit do not assign gardeners their own garden plot to cultivate; instead, the entire community garden is typically cultivated collectively. The size of Garden Resource Program member gardens varies widely. For example, in 2014 about 25% of gardens were smaller than 100 square feet, about 21% were between 100 and 400 square feet, 24% were between 400 square feet and one city lot, and about 30% were larger than one city lot [31].

### 2.2. Research Methods

This research used a community-based participatory research approach. A research committee comprised of Keep Growing Detroit staff, Garden Resource Program members, and academic researchers oversaw the project’s design and implementation. The committee chose semi-structured qualitative interviews as the mode of data collection to obtain in-depth accounts of participants’ experiences. The research committee chose topics to include in the interview guide, and the principal investigator designed open-ended questions based on these topics. The research committee provided feedback on the questions, and the principal investigator revised the interview guide to incorporate this feedback prior to data collection. The interview guide contained questions regarding the impact of gardening on mental and physical health and wellbeing (see Supplementary Materials). The interviews also examined the nutritional impacts of gardening, which have been published elsewhere [32]. This research study was approved by the Michigan State University Institutional Review Board (protocol #14-110).

The interviews were conducted in 2015 by three research committee members: the principal investigator and two Garden Resource Program members. The principal investigator trained the interviewers in qualitative interview techniques prior to data collection. Twenty-eight Garden Resource Program members were interviewed, and participants were chosen through purposive sampling by the research committee to mirror GRP members in terms of their type of garden, age, race, and gender. Interviews were tape-recorded and transcribed, and the transcripts were checked for accuracy. Interviews were coded in Atlas.ti using a combined inductive–deductive thematic coding approach. An initial codebook was generated that included deductive codes based on the interview guide questions and inductive codes identified during transcript checking and from nine randomly chosen interviews. Next, two trained researchers each coded an additional nine interviews using this initial codebook. The two coders discussed coding differences, refined the codebook, and reached a consensus on coding. The first author coded the remaining interviews with this refined codebook. Data saturation was reached and was determined by the absence of new emergent themes found in the interviews. After the coding of all interviews was completed, codes were clustered into larger themes related to the primary research questions: physical health, physical activity, and mental wellbeing. For each of these themes, summary statements were written for each gardener and arranged in conceptually-clustered matrix displays [33]. Conclusions were drawn by identifying patterns across participants.

## 3. Results

### 3.1. Characteristics of Participants

The participant demographics are provided in Table 1. There were equal numbers of male and female participants. Most participants identified as African American and were aged over 50.

### 3.2. Physical Activity and Physical Health

Nearly half of the participants described gardening as physically demanding. Gardeners emphasized the physical work of gardening, either implicitly or explicitly stating that gardening was good exercise. For example, participant 5 said “Of course I’m not jogging, but I’m working. Swinging a hoe for several hours a day is no joke, or hands and knees weeding”.

Similarly, several other participants mentioned specific gardening tasks that were especially physically strenuous, including hauling compost, carrying buckets of water, and digging. The wide variety of tasks needed to tend a garden, as well as the variety of types of movement (bending, stretching, and lifting), were described as working many parts of the body, with one gardener saying, “You use every muscle in your body. You’re using your back, your arms, your knees, your thighs, your calves, your ankles, your wrists, your elbows” (participant 12). Similar sentiments were shared by gardeners of all ages, and by gardeners with and without physical limitations. For example, one participant stated, “So I’d be riding my scooter up and down the lanes leaning out weeding… so that’s one thing you can say about gardening. It’s exercise” (participant 1). Further evidence for the intensity of physical activity during gardening came from participants who reported needing to stay in shape to be able to garden. This need encouraged some participants to exercise outside of the garden in ways that would improve their fitness for gardening. One participant also described a neighborhood walking group that had formed around the garden: “We walk in the neighborhood, check the garden out, watch out for the kids” (participant 11).

Gardening was a primary source of physical activity for some participants. These participants engaged in little to no intentional exercise outside of the garden and they preferred gardening to more structured forms of exercise, such as going to the gym or running. As one gardener said, “Well, it gets me outside. And off my butt. I don’t like to exercise… so gardening is really my only exercise” (participant 15). Others also reported disliking other forms of exercise, indicating that gardening provided a source of physical activity for people who would not participate in much activity otherwise. In fact, other participants explained that they had been inactive before gardening and that the activity from gardening benefited their bodies. As one gardener said, “When I started gardening, it helped my body. Yeah, gardening makes you work. … for couple of years [before gardening] I was a couch potato” (participant 2). The importance of the activity from gardening was evident when participants compared their physical activity level during the gardening season with the winter. During the winter, gardeners mentioned that their activity levels were drastically lower and that they missed the physical activity from the garden. For example, one gardener said, “In the winter time I am lost because I don’t have that exercise that I get out of being in the garden” (participant 14).

The physical activity from the garden was perceived to improve physical function, particularly for participants aged over 50 who had physical limitations or physical pain. These gardeners reported improvements in arthritis symptoms, increased flexibility, and regaining physical function after an accident. One gardener said, “My therapeutic gardening, I call it because the knee problems and the back problems and the arthritis, and all that, seems to be better because of my movement” (participant 14). Another gardener who had lost function in his hand after an accident credited gardening with regaining hand function. He said,

*The doctors told me that that’s all we can do for you. We can’t do anymore. I said well I know what to do. I will go back to gardening which is something that will work my body and work my hand and I have dug that garden across the street with my hands.*

Participants attributed a variety of other physical health improvements to gardening. Both improved dietary habits, such as eating more fruits and vegetables (described in detail in Beavers et al. [32]), and physical activity from gardening were credited with these health improvements. The perceived physical health benefits included improvements in chronic diseases, such as reduced blood sugar, reduced blood pressure, or cancer remission. Additionally, some gardeners also reported that gardening had caused them to lose weight or prevented weight gain. As participant 14 explained, “In the winter time I have a real hard problem regulating my weight. Summer time when the garden is open I don’t have any problem because for two reasons, I am actually working and I am actually eating healthy”. Eating garden vegetables and being active in the garden were also credited with making the participants feel better physically. For example, some interviewees reported that they had more energy because of these aspects of gardening, which allowed them to be more active outside of the garden.

Two participants credited gardening with staying healthy while aging. As one participant said, “I don’t catch colds. And I haven’t had any type of problems, high blood pressure, nothing. I don’t take any pills, and I’m collecting social security now…I think working in the garden has a whole lot to do with” (participant 2). Gardeners also reported that spending more time outside, receiving vitamin D from the sun, and better sleep were health benefits of gardening.

### 3.3. Stress Relief and Improved Mood

Stress relief and relaxation provided by gardening were prevalent themes related to mental wellbeing. Participants used words such as “peace”, “calm”, “grounded”, and “refreshed” to describe how gardening influenced their mental state. Several aspects of gardening were important for relieving stress and promoting relaxation. Some gardeners attributed stress relief to the physical act of gardening, tying the physical activity from the garden to this benefit. As participant 1 said, “It was just a true release of the stress ‘cause there was a lot of work to do [in the garden]… So it sort of like stimulate your brain and make it relaxed.” Interacting with nature by tending to the garden’s plants was another way in which the act of gardening contributed to stress relief: “… being able to interact and help cultivate the growth of such a plant and interact with life is a really different… is a very calm state when I am out in the garden” (participant 17). Participants also described the garden as a peaceful place. Some noted that just looking at the garden was relaxing, while others described a sense of peace or calm that they felt from just being in the garden: “I notice that in that little area where the garden is… it seems to be a peace…” (participant 10). The garden space contributed to stress relief by offering an opportunity to escape or being away from the stressors of daily life, with gardeners describing the garden as being separate from the outside world. This physical separation allowed gardeners to mentally disengage from the stress of their daily lives: “When I come home from work I will go straight to my garden and I don’t even think about my job anymore. It’s just–it’s another world” (participant 4). For another gardener, interacting with plants was closely tied to the garden being an escape: “What I enjoy is the life that it brings to each day…if I keep my focus on my relationship with the seeds and the plants, it’s almost like it’s a world that the world can’t invade…” (participant 13).

Participants also explained that gardening improved their mood by making them happy or bringing them joy, as well as by reducing negative emotions, such as anger or sadness. Some of the same aspects of gardening that were linked to stress relief were important for improvements in mood: the garden being a happy place and the interaction with plants. Just as being in the garden was reported to relieve stress, gardeners also described how the physical garden space made them feel happy. As one gardener said, “I cannot tell you how great I feel when I am in the midst of that garden…” (participant 10). Another participant said, “Even just seeing my husband work it and the joy that he gets out of it is joy for me… just the presence of it is very comforting to me” (participant 16). Other gardeners reported that gardening provided an opportunity to help others, which is described in more detail below. Tending to or even just being with plants was another way that gardening made people happy or improved their mood. As gardener 8 said, “I always feel better, if I’m angry or upset, go be with the plants, talk to the plants, listen to the plants, I feel better”. Some also talked about how the garden helped them cope with difficulties in their lives, calling the garden “therapeutic”. After the death of her son, one gardener said,

*It woke me up. As I said, after my son passed away I was very depressed. I had to heal mentally and physically. [Gardening] was something I was able to do that I was able to do. It brought satisfaction to me, when I didn’t feel like eating, sleeping. So, that was something, a new day, that I can look forward. It’s therapy for me. It’s therapeutic in many ways… with the soul, the body (participant 3).*

### 3.4. Helping Others and Personal Growth

Gardening was perceived to impact the participants beyond mental and physical health. Many gardeners described how gardening contributed to their personal growth through helping others and in other ways. For instance, some gardeners explained that they helped others by sharing their garden harvest or sharing gardening knowledge and inspiring others to start gardens. As participant 2 said, “I tend to want to help people more [since gardening], because people started coming to me, asking questions… three to four people have kicked off some really nice gardens”. Helping others also benefited the gardeners themselves: gardeners expressed feelings of happiness that came from helping others through the garden. For example, participant 4 said, “I have to do everything that I can to…share with others and give to others that don’t have…That’s very, very important to me”. Gardeners also described how gardening has contributed to their personal growth in ways beyond helping others. Several gardeners described how gardening made them grow by becoming more patient, tolerant, or laidback. Other gardeners experienced personal growth by learning lessons from observing the natural world and applying these lessons to their own lives. As one gardener said, “I gained, knowledge, I gained knowing how to live life… I learned how to just sit down and you know smell the flowers, look at the flowers, look at the sky, look at the birds” (participant 11). Another participant used examples of natural processes to metaphorically apply them to his life. This gardener said,

*I see how God is able to take and recycle a seed and take a broken, maybe wounded plant and… that plant just takes off and then the rest of it becomes beautiful all over again. Such as life. Take off that attitude, whether it be drinking, sex, gambling, or a mental attitude, and take it off and then you can begin to flourish… What can happen to a seed, it can happen to a human heart and a human life as well (participant 13).*

This quote is also an example of how gardening was related to spirituality or religion, which will be described in more detail in the next section.

### 3.5. Spirituality and Religion

Most gardeners indicated that there was a relationship between gardening and their religion or spirituality. This relationship was present for participants who self-identified as being religious, as well as for participants who reported not being religious. For those that were religious, gardening reinforced or deepened their religious beliefs, such as by providing an opportunity to connect to God. As one gardener said, “Being in the garden just really quiets me down, puts me in contact with God, that’s right, that’s my prayer time really…I have learned to relate to God in a much different way and much more immediate [way]” (participant 15). Several other gardeners described the garden as God’s gift or creation.

The exposure to nature in the garden was the most commonly cited factor that linked the garden to religion or spirituality. Several gardeners used terms such as being “in tune with nature” or “one with nature” in the garden to articulate this connection. The garden soil, birds, bees, and plants were all components of nature that were linked to spirituality and religion. An example of this was provided by participant 10: “Put your feet in that dirt and put your hand in that dirt, you will find Him, He’s right there in that soil”. Another participant said, “Well the spiritual dimension, it’s just amazing. The spiritual link between plants and humans and how the behavior of humans can impact the behavior of plants” (participant 7). Additionally, gardening was referred to as a part of “culture” or as a “tradition;” a way to connect people across generations. Some gardeners described gardening as a way to connect with their ancestors; as one gardener stated, “I mean [gardening is] so much a part of what my family has done. What is in African-Americans, from our traditions… it was just a part of the culture” (participant 16). Others talked about their desire to keep the next generation gardening: “So, we must keep this tradition alive. And continue it, and pass it on” (participant 3).

## 4. Discussion

This study qualitatively examined the perceived health and well-being impacts of urban gardening in a primarily African American sample of urban gardeners belonging to a gardener support program. Overall, our findings were highly consistent with previous research with less racially diverse participants conducted in Western countries. This study provides supporting evidence for the role of gardening in improving health in diverse populations.

Several gardeners in our study described gardening as physically demanding and that it worked many parts of their bodies. In other qualitative studies, gardeners also described gardening as an all-around exercise, or that it worked the muscles by requiring a wide variety of activities [26,27]. Some gardeners in our study reported that gardening was their main source of physical activity, and that they preferred gardening to more traditional forms of exercise, such as working out in a gym. This preference for physical activity through gardening was found in several other qualitative studies with all white or majority white participants [25,26,27]. Additionally, some gardeners in our study reported that they were previously inactive before gardening. This indicates that gardening may be an enjoyable way for people to become active who would not engage in much intentional physical activity otherwise. However, most quantitative research examining differences in physical activity between gardeners and non-gardeners or examining changes in physical activity after gardening does not support the notion that gardening increases physical activity [12,13,18,19,20]. These discrepancies between qualitative and quantitative literature warrant further investigation.

In this study, the physical health improvements that participants attributed to gardening included improvements in physical function, such as increased flexibility or reductions in arthritis symptoms, and improvements in chronic diseases, including hypertension and diabetes. Similarly, other qualitative research has found that urban gardeners attribute a variety of physical health improvements to gardening, ranging from weight loss, maintaining health while aging, and feeling more fit [26,27,30]. There is currently scant objective data examining the relationship between gardening and physical health, and many of the studies to date have been small pilot studies. However, the small amount of existing evidence suggests that gardening may benefit physical health. In cross-sectional studies of older adults, gardeners had higher hand strength and pinch force than non-gardeners, better balance, and reduced risk of falls [34,35]. Two pilot randomized controlled trials also examined the impact of gardening on objective measures of physical function in cancer survivors. One of these studies found that gardeners exhibited significant improvement in three out of nine measures of physical performance, while the other found that gardeners exhibited significant improvement in six out of seven measures of physical performance [12,13].

Mental health benefits, such as stress relief, relaxation, and improved mood, were commonly mentioned in this study. Gardeners attributed these benefits to several aspects of gardening, including the act of gardening tasks and the garden itself as a peaceful place to be. Several other studies have similarly found that both the active and passive aspects of gardening were reported to be beneficial for mental wellbeing [25,27,36]. Related to mental wellbeing, the gardeners described many ways in which they experienced nature in the garden, actively through tending the plants and passively through observing the sights and sounds. Additionally, the natural landscape of the garden provided an opportunity to escape from daily urban life. The mental benefits of nature contact in the garden and the garden as an “escape” were found in many other studies of urban gardeners [25,26,27,28]. There is also quantitative evidence to support the association between gardening and mental well-being. A case–control study comparing allotment gardeners and matched non-gardeners in England found that allotment gardeners had significantly better self-esteem, general mental health, depression, vigor, and fatigue, as well as composite score of total mood disturbance [37]. This study also assessed the same metrics in gardeners before and after a session of allotment gardening. After the gardening session, the gardeners had significantly improved self-esteem, tension, depression, anger, confusion, and composite total mood disturbance score [37]. Additionally, a randomized controlled trial objectively measured the impact of gardening on stress by measuring the stress hormone cortisol. Community gardeners conducted a mentally stressful task and were then randomly assigned to either conduct light gardening activities or read indoors. Those who were assigned to gardening had a larger reduction in cortisol after the stressful task [21].

The key strengths of this work include the participatory planning process, qualitative design, and the diversity of the participants. Community-based participatory research can illuminate unique place-based circumstances that can impact research findings. Having Detroit gardeners co-write research questions, the interview guide, and conduct interviews facilitated trust and engagement among the research participants. Although the reported physical activity and physical health benefits of gardening were not objectively measured, the qualitative design allowed for the first-hand experiences and perceptions of the gardeners to directly identify the many ways in which gardening is perceived to influence physical and mental health. The qualitative design also allowed for in-depth investigation into which aspects of gardening are perceived to influence health. Additionally, this is one of the few studies on urban gardening and health in which the majority of participants are people of color. Hearing from diverse voices allows for a comprehensive representation of the range of meaning and experiences related to gardening and health, including the distinctive spiritual nature of gardening providing meaningful connections to their origin and ancestors.

## 5. Conclusions

In conclusion, this study was one of few to examine the perceived health impacts of urban gardening in a racially diverse sample of participants in the United States. Participants commonly reported that gardening was beneficial to physical activity, physical health, and mental wellbeing, which is highly consistent with previous qualitative research conducted with mostly white participants in Western countries, but also shared their unique perspectives from their own cultures and communities. Our findings demonstrate that gardening has the potential to influence health in diverse groups of people, and thus may have the potential to address health disparities.

## Figures and Tables

**Table 1:** Demographic characteristics of participants ^1^.

	Number of Participants
Gender	
Female	13
Male	13
Race	
White	4
Black or African American	17
Hispanic or Latino/a	1
American Indian or Alaskan Native	1
Multiple races or other	3
Age in years	
<18	1
31–40	2
41–50	2
51–65	12
66+	8

^1^ Participants were free to not answer any question they wished not to answer. Two participants declined to complete the questionnaire and one additional participant declined to provide their age.

## Data Availability

Not applicable (qualitative research).

## Supplementary Materials

The following supporting information can be downloaded at: https://www.mdpi.com/article/10.3390/ijerph19137899/s1, File S1: INTERVIEW GUIDE.

## References

[1] Moore L.V., Dodd K.W., Thompson F.E., Grimm K.A., Kim S.A., Scanlon K.S. (2015). Using Behavioral Risk Factor Surveillance System Data to Estimate the Percentage of the Population Meeting US Department of Agriculture Food Patterns Fruit and Vegetable Intake Recommendations. Am. J. Epidemiol..

[2] National Center for Health Statistics FastStats: Exercise or Physical Activity.

[3] Wang P.Y., Fang J.C., Gao Z.H., Zhang C., Xie S.Y. (2016). Higher Intake of Fruits, Vegetables or Their Fiber Reduces the Risk of Type 2 Diabetes: A Meta-Analysis. J. Diabetes Investig..

[4] Wang X., Ouyang Y., Liu J., Zhu M., Zhao G., Bao W., Hu F.B. (2014). Fruit and Vegetable Consumption and Mortality from All Causes, Cardiovascular Disease, and Cancer: Systematic Review and Dose-Response Meta-Analysis of Prospective Cohort Studies. BMJ.

[5] Aune D., Norat T., Leitzmann M., Tonstad S., Vatten L.J. (2015). Physical Activity and the Risk of Type 2 Diabetes: A Systematic Review and Dose–Response Meta-Analysis. Eur. J. Epidemiol..

[6] Wahid A., Manek N., Nichols M., Kelly P., Foster C., Webster P., Kaur A., Friedemann Smith C., Wilkins E., Rayner M. (2016). Quantifying the Association Between Physical Activity and Cardiovascular Disease and Diabetes: A Systematic Review and Meta-Analysis. J. Am. Heart Assoc..

[7] National Institute of Mental Health (NIMH) Mental Illness. https://www.nimh.nih.gov/health/statistics/mental-illness.

[8] Alaimo K., Beavers A.W., Crawford C., Snyder E.H., Litt J.S. (2016). Amplifying Health Through Community Gardens: A Framework for Advancing Multicomponent, Behaviorally Based Neighborhood Interventions. Curr. Environ. Health Rep..

[9] Alaimo K., Packnett E., Miles R.A., Kruger D.J. (2008). Fruit and Vegetable Intake among Urban Community Gardeners. J. Nutr. Educ. Behav..

[10] Litt J.S., Soobader M.J., Turbin M.S., Hale J.W., Buchenau M., Marshall J.A. (2011). The Influence of Social Involvement, Neighborhood Aesthetics, and Community Garden Participation on Fruit and Vegetable Consumption. Am. J. Public Health.

[11] Barnidge E.K., Hipp P.R., Estlund A., Duggan K., Barnhart K.J., Brownson R.C. (2013). Association between Community Garden Participation and Fruit and Vegetable Consumption in Rural Missouri. Int. J. Behav. Nutr. Phys. Act..

[12] Bail J.R., Fruge A.D., Cases M.G., De Los Santos J.F., Locher J.L., Smith K.P., Cantor A.B., Cohen H.J., Demark-Wahnefried W. (2018). A Home-Based Mentored Vegetable Gardening Intervention Demonstrates Feasibility and Improvements in Physical Activity and Performance among Breast Cancer Survivors. Cancer.

[13] Demark-Wahnefried W., Cases M.G., Cantor A.B., Fruge A.D., Smith K.P., Locher J., Cohen H.J., Tsuruta Y., Daniel M., Kala R. (2018). Pilot Randomized Controlled Trial of a Home Vegetable Gardening Intervention among Older Cancer Survivors Shows Feasibility, Satisfaction, and Promise in Improving Vegetable and Fruit Consumption, Reassurance of Worth, and the Trajectory of Central Adiposity. J. Acad. Nutr. Diet..

[14] Carney P.A., Hamada J.L., Rdesinski R., Sprager L., Nichols K.R., Liu B.Y., Pelayo J., Sanchez M.A., Shannon J. (2012). Impact of a Community Gardening Project on Vegetable Intake, Food Security and Family Relationships: A Community-Based Participatory Research Study. J. Community Health.

[15] Park S.A., Lee K.S., Son K.C. (2011). Determining Exercise Intensities of Gardening Tasks as a Physical Activity Using Metabolic Equivalents in Older Adults. Hortscience.

[16] Park S.A., Lee A.Y., Lee K.S., Son K.C. (2014). Gardening Tasks Performed by Adults Are Moderate- to High-Intensity Physical Activities. HortTechnology.

[17] Center for Disease Control and Prevention How Much Physical Activity do Adults Need?.

[18] Soga M., Cox D.T., Yamaura Y., Gaston K.J., Kurisu K., Hanaki K. (2017). Health Benefits of Urban Allotment Gardening: Improved Physical and Psychological Well-Being and Social Integration. Int. J. Environ. Res. Public Health.

[19] Hawkins J.L., Thirlaway K.J., Backx K., Clayton D.A. (2011). Allotment Gardening and Other Leisure Activities for Stress Reduction and Healthy Aging. HortTechnology.

[20] van den Berg A.E., van Winsum-Westra M., de Vries S., van Dillen S.M. (2010). Allotment Gardening and Health: A Comparative Survey among Allotment Gardeners and Their Neighbors without an Allotment. Environ. Health A Glob. Access Sci. Source.

[21] Van Den Berg A.E., Custers M.H. (2011). Gardening Promotes Neuroendocrine and Affective Restoration from Stress. J. Health Psychol..

[22] Wendelboe-Nelson C., Kelly S., Kennedy M., Cherrie J.W. (2019). A Scoping Review Mapping Research on Green Space and Associated Mental Health Benefits. Int. J. Environ. Res. Public Health.

[23] Shuda Q., Bougoulias M.E., Kass R. (2020). Effect of Nature Exposure on Perceived and Physiologic Stress: A Systematic Review. Complement. Ther. Med..

[24] Twohig-Bennett C., Jones A. (2018). The Health Benefits of the Great Outdoors: A Systematic Review and Meta-Analysis of Greenspace Exposure and Health Outcomes. Environ. Res..

[25] Hale J., Knapp C., Bardwell L., Buchenau M., Marshall J., Sancar F., Litt J.S. (2011). Connecting Food Environments and Health through the Relational Nature of Aesthetics: Gaining Insight through the Community Gardening Experience. Soc. Sci. Med..

[26] Kingsley J.Y., Townsend M., Henderson-Wilson C. (2009). Cultivating Health and Wellbeing: Members’ Perceptions of the Health Benefits of a Port Melbourne Community Garden. Leis. Stud..

[27] Hawkins J.L., Mercer J., Thirlaway K.J., Clayton D.A. (2013). “Doing” Gardening and “Being” at the Allotment Site: Exploring the Benefits of Allotment Gardening for Stress Reduction and Healthy Aging. Ecopsychology.

[28] Wakefield S., Yeudall F., Taron C., Reynolds J., Skinner A. (2007). Growing Urban Health: Community Gardening in South-East Toronto. Health Promot. Int..

[29] Kortright R., Wakefield S. (2011). Edible Backyards: A Qualitative Study of Household Food Growing and Its Contributions to Food Security. Agric. Hum. Values.

[30] Palar K., Lemus Hufstedler E., Hernandez K., Chang A., Ferguson L., Lozano R., Weiser S.D. (2019). Nutrition and Health Improvements After Participation in an Urban Home Garden Program. J. Nutr. Educ. Behav..

[31] Beavers A.W., Atkinson A., Ma W., Alaimo K. (2021). Garden Characteristics and Types of Program Involvement Associated with Sustained Garden Membership in an Urban Gardening Support Program. Urban For. Urban Green..

[32] Beavers A., Atkinson A., Alaimo K. (2019). How Gardening and a Gardener Support Program in Detroit Influence Participants’ Diet, Food Security, and Food Values. J. Hunger Environ. Nutr..

[33] Miles M.B., Huberman A.M., Saldana J. (2014). Qualitative Data Analysis: A Methods Sourcebook.

[34] Park S.A., Shoemaker C.A., Haub M.D. (2009). Physical and Psychological Health Conditions of Older Adults Classified as Gardeners or Nongardeners. Hortscience.

[35] Chen T.-Y., Janke M.C. (2012). Gardening as a Potential Activity to Reduce Falls in Older Adults. J. Aging Phys. Act..

[36] Milligan C., Gatrell A., Bingley A. (2004). ‘Cultivating Health’: Therapeutic Landscapes and Older People in Northern England. Soc. Sci. Med..

[37] Wood C.J., Pretty J., Griffin M. (2016). A Case-Control Study of the Health and Well-Being Benefits of Allotment Gardening. J. Public Health.

