# Supplementary material for: How Gardening in Detroit Influences Physical and Mental Health
Source: Int J Environ Res Public Health. 2022 Jun 28;19(13):7899. doi: 10.3390/ijerph19137899 (PMC9265422; doi:10.3390/ijerph19137899)
Supplement: Supplementary file 1 [file ijerph-19-07899-s001.zip › ijerph-1652621-supplementary.pdf]

INTERVIEW GUIDE

1. Can you share with me some of your recent or memorable gardening and farming experiences?
2. Why do you garden [or farm] and what do you enjoy?
3. What keeps you involved in gardening [or farming]?
4. How has gardening or your garden impacted your life?
5. Has your physical activity or exercise habits changed because of the garden? [If they say yes]: How has your activity changed because of the garden?
6. Have there been any changes in your health or things that you do to be healthy that you could attribute to gardening? [If they say yes]: What are they?
7. Has gardening affected your mood, stress level, or sleep? [If they say yes]: How?
8. Has gardening changed how you feel about spirituality? [If they say yes]: How?
